# Cross-frequency transfer in a stochastically driven mesoscopic neuronal model

**DOI:** 10.3389/fncom.2015.00014

**Published:** 2015-02-16

**Authors:** Maciej Jedynak, Antonio J. Pons, Jordi Garcia-Ojalvo

**Affiliations:** ^1^Departament de Física i Enginyeria Nuclear, Universitat Politècnica de CatalunyaBarcelona, Spain; ^2^Department of Experimental and Health Sciences, Universitat Pompeu Fabra, Parc de Recerca Biomèdica de BarcelonaBarcelona, Spain

**Keywords:** cross-frequency coupling, stochastic, neural mass model, Jansen-Rit model, neuronal oscillations, driven oscillators, mesoscopic brain dynamics, Ornstein-Uhlenbeck noise

## Abstract

The brain is known to operate in multiple coexisting frequency bands. Increasing experimental evidence suggests that interactions between those distinct bands play a crucial role in brain processes, but the dynamical mechanisms underlying this cross-frequency coupling are still under investigation. Two approaches have been proposed to address this issue. In the first one distinct nonlinear oscillators representing the brain rhythms involved are coupled actively (bidirectionally), whereas in the second one the oscillators are coupled unidirectionally and thus the driving between them is passive. Here we elaborate the latter approach by implementing a stochastically driven network of coupled neural mass models that operate in the alpha range. This model exhibits a broadband power spectrum with 1/*f^b^* form, similar to those observed experimentally. Our results show that such a model is able to reproduce recent experimental observations on the effect of slow rocking on the alpha activity associated with sleep. This suggests that passive driving can account for cross-frequency transfer in the brain, as a result of the complex nonlinear dynamics of its underlying oscillators.

## 1. Introduction

Brain activity, as registered in macroscopic recordings like EEG, does not yield power spectra composed of multiple isolated and narrow frequency peaks, but of broad frequency bands that are merged with each other in a seamless manner, and which are embedded in a 1/*f^b^* background with large power at low frequencies and a fat tail at high frequencies (Freeman et al., 2000; Buzsáki and Draguhn, 2004). Nonlinearities in the interactions between neuronal populations (Friston, 2000) can be expected to lead to mixing within this continuum of frequencies. Indeed, transcranial stimulation of the brain at low frequencies (smaller than 1 Hz) has been seen to cause for instance an increase in oscillatory power at larger frequencies (5–10 Hz) (Marshall et al., 2006; Massimini et al., 2007). Much emphasis has been placed recently on quantifying and characterizing the transfer of spectral power across frequencies (known in what follows as cross-frequency coupling, Jirsa and Müller, 2013), and on identifying its functional roles in the brain (Jensen and Colgin, 2007; Canolty and Knight, 2010). In particular, power spectrum correlations have been observed for instance between theta and gamma rhythms in the rat hippocampus during memory retrieval (Shirvalkar et al., 2010), between posterior gamma and frontal alpha/beta oscillations in the human brain during motor imagery tasks (de Lange et al., 2008), and between the gamma and delta bands in different regions of the human visual cortex during a visual task (Bruns and Eckhorn, 2004). Phase coupling between delta and alpha bands has also been reported in human brains performing an orientation task (Isler et al., 2008). More common is the situation in which the oscillation power in a given frequency band is modulated by a second rhythm at lower frequency. Such phase-to-amplitude cross-frequency coupling has been observed for instance between alpha and gamma activities in humans during rest (Osipova et al., 2008) and between theta and gamma oscillations in rats during learning (Tort et al., 2009). Other behavioral correlates of cross-frequency coupling have been found, associated for instance with reward coding (Cohen et al., 2009a) and decision making (Cohen et al., 2009b) in humans. Also, recent work has shown that such cross-frequency coupling is modulated by behavioral tasks (Voytek et al., 2010). Cox et al. (2014) reported cross-frequency coupling between the phase of sleep spindles and the amplitude of higher frequency rhythms, in particular beta, recorded in EEG during sleep. This effect, in turn, was modulated in the frontal cortex by the phase of slow sleep oscillations.

Despite the large number of experimental studies pointing toward cross-frequency correlations, several difficulties arise when it comes to the interpretation of this phenomenon. As pointed out by Aru et al. (2015), the methodologies applied in a number of recent studies on cross-frequency coupling are not flawless and the results might have been overinterpreted. Therefore, further and stricter studies on the functional role of cross-frequency coupling are needed to confirm previous results. In particular, not all cross-frequency correlations are signatures of direct interaction between rhythms. When they are, such correlations may be explained by different mechanisms, which may be grouped into two broad scenarios. In one scenario, two neuronal oscillators operating at two different rhythms might be coupled bidirectionally to each other. This coupling could mediate an interaction that would result in each of the oscillators being affected in one way or another by the natural frequency of the other oscillator (Jirsa and Müller, 2013). When such bidirectional interaction occurs locally, it has been proposed to be mediated by the firing activity of the underlying neurons (Mazzoni et al., 2010). In such a way delta oscillations, for instance, control the level of local cortical excitability, which in turn modulates the excitatory-inhibitory balance that gives rise to the gamma rhythm (Mazzoni et al., 2011). In a second, somewhat simpler scenario, cross-frequency correlations might arise due to unidirectional coupling, through which the spectral features of the driving neuronal population would be directly transferred to the driven population. When the same external stimulus is encoded by two different rhythms, cross-frequency correlations can appear as a result of that common unidirectional driving (Mazzoni et al., 2008). In some cases, however, the stimulus does not necessarily affect directly the neurons underlying one of the rhythms. This might be the case of recent experimental work by Bayer et al. (2011), who examined the effect of rocking on sleep in human subjects. In that study, healthy volunteers were asked to lie down on a rocking bed that oscillated slowly, at a frequency of 0.25 Hz. This periodic stimulation was seen to ease the transition from waking to sleep, and to increase the power of cortical oscillations (measured via EEG) in the alpha range. Here we ask whether a cross-frequency transfer such as that reported by Bayer et al. (2011) can be the result of the low frequency input driving a mesoscopic broadband oscillator operating in the alpha range. To that end, we need a mesoscopic model of brain activity.

Brain dynamics at the mesoscale is frequently described by population models such as the neural mass model (NMM), originating from the works of Freeman (1972), Wilson and Cowan (1972), Amari (1974), Lopes da Silva et al. (1974), and Nunez (1974). These models aim to reproduce the average behavior of relatively large populations of cells. The dynamical unit in this model can be interpreted as a cortical hypercolumn (Jansen et al., 1993; Jansen and Rit, 1995), the model variables being the average postsynaptic potentials of the different neuronal populations (Faugeras et al., 2009). NMMs have been extensively used in recent years to describe a wide variety of brain behaviors including rhythm generation (Ursino et al., 2010) and propagation (Cona et al., 2011), spontaneous dynamics (Nguyen Trong et al., 2012), photic stimulation (Spiegler et al., 2011), criticality (Aburn et al., 2012), and even plasticity (Wang and Knösche, 2013). Aberrant dynamics in epilepsy has been described with NMMs both at the level of the generation (Wendling et al., 2000) and termination (Goodfellow et al., 2012; Freestone et al., 2013) of epileptic seizures. Coupled NMMs have also been used to examine the relationship between structural and functional connectivity in healthy and neurodegenerative conditions (Pons et al., 2010; Ponten et al., 2010). Generalizing the NMM description to continuous space gives rise to neural field models (Jirsa and Haken, 1996; Coombes, 2010), which have been used to study the spatiotemporal dynamics of cortical waves (Hutt and Atay, 2006; Bojak and Liley, 2010) and even to implement control strategies in robotics (Erlhagen and Bicho, 2006).

NMMs can be made to exhibit a variety of rhythms depending on the values of the dynamic parameters (David and Friston, 2003), but in most situations the oscillations obtained are relatively narrowband. This contrasts with the 1/*f^b^* spectra usually observed experimentally (Freeman et al., 2000). In neural fields, such type of spectral behavior has been linked to the multiple spatial scales characteristic of spatially extended neuronal tissue, where it is observed near a stability threshold (Hutt and Frank, 2005). In this paper, we reproduce the spectral properties measured by Bayer et al. (2011) by applying a temporally correlated noise to a coupled NMM. This is a reasonable assumption, since the brain has multiple sources of noise (Faisal et al., 2008) that have a variety of functional roles (McDonnell and Ward, 2011). In microscopic models, a temporally correlated Ornstein-Uhlenbeck noise is known to reproduce the observed 1/*f* spectral profile of LFP activity (Sancristóbal et al., 2013). Our results show that a network of coupled neural masses subject to temporally correlated noise exhibits a well-defined rhythm (in the alpha range) embedded in a broadband spectral background similar to what is observed experimentally. We also show that this broadband oscillator reacts to periodic driving at a frequency much lower than its natural frequency, by increasing its activity at the latter in agreement with experimental observations.

## 2. Materials and methods

### 2.1. Extending Jansen and Rit model

The basic building block of our model is a cortical column (Hubel and Wiesel, 1977; Helmstaedter et al., 2007; Ts'o et al., 2009) that we describe in the way proposed by Jansen et al. (1993). In this model the neurons of a cortical column are classified into three neuronal populations: pyramidal neurons, excitatory interneurons, and inhibitory interneurons. The dynamics of each population is described using two simple transformations. The first one stands for synaptic processing: it describes how the presynaptic signal coming from interconnected populations translates into a postsynaptic membrane potential. This transformation is linear and is given by the convolution:

(1) $$\begin{array}{l} y(t)=\int_{-\infty }^{t}h(t^{\prime })p_{\text{tot}}(t-t^{\prime })\text{d}t^{\prime }, \end{array}$$

where *p*_tot_(*t*) is a total input acting upon the population, expressed in terms of a firing rate, *y*(*t*) is the net postsynaptic membrane potential, and *h*(*t*) is an impulse response. The kernel of the transformation is valid for *t* > 0 and is defined for excitatory and inhibitory connections as follows:

(2) $$h_{e}(t)\;=Aate^{-at}$$

(3) $$\;h_{i}(t)=Bbte^{-bt},$$

where *A* and *B* are the maximum excitatory and inhibitory postsynaptic potential amplitudes, respectively, and *a* and *b* are inverse time constants that lump together all signal propagation delays. This transformation can be expressed in differential form using the Laplace transform, which leads to:

(4) $$\frac{d^{2}y(t)}{dt^{2}}+2a\frac{dy(t)}{dt}+a^{2}y(t)=Aa\cdot p_{\text{tot},\text{exc}}(t),$$

where *y*(*t*) is a postsynaptic membrane potential averaged over all neurons in a given population, and *p*_tot, exc_ is the sum of firing rates of all excitatory signals coming into that population. An equivalent expression containing the sum of all incoming inhibitory signals *p*_tot, inh_(*t*) with constants *B* and *b* describes inhibitory processing.

The second transformation describes how the postsynaptic membrane potential within a population translates into a firing rate in that population's output. This transformation is nonlinear and is given by:

(5) $$\text{ Sigm}(y)=\frac{2e_{0}}{1+e^{r(\nu_{0}-y)}},$$

where 2*e*_0_ is the maximum firing rate, ν_0_ is the potential for which the firing rate is equal to half of the maximum, and *r* determines the steepness (and nonlinearity) of the response.

**Figure 1:**
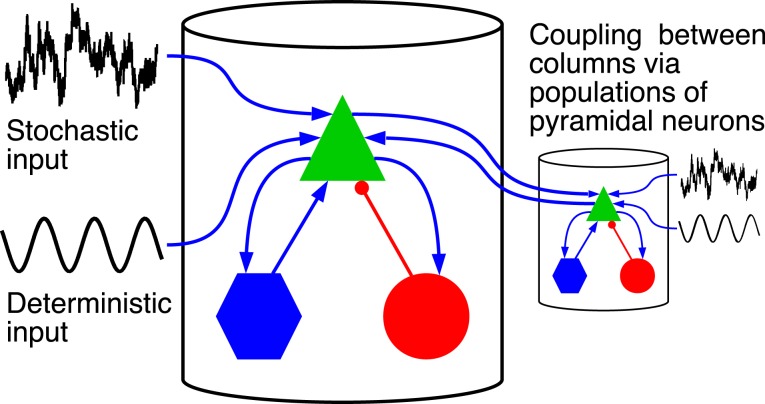
**Driving inputs and connectivity between neuronal populations within a single cortical column and between columns**. The model is formed by a network of coupled cortical columns described by the Jansen-Rit model. In each column a population of pyramidal neurons (green triangle), feeds forward to a population of excitatory interneurons (blue hexagons) and inhibitory interneurons (red circles). The two interneuron populations feed back into the pyramidal neurons. Excitatory connections are marked with blue arrows, and inhibitory connection with red lines with circular endings. The model is homogeneous; all columns are identical and all inter-column connections have same strength. Columns are fed with a common deterministic input and independent realizations of an Ornstein-Uhlenbeck noise. The deterministic input denoted here by a sinusoid has a constant DC level.

Figure 1 shows a schematic representation of the model. It is built as a network of coupled cortical columns. Within a single column a population of pyramidal neurons feeds forward to the excitatory and inhibitory interneuron populations, which in turn feed back to the pyramidal neurons leading to positive and negative feedback, respectively. The excitatory and inhibitory interneurons receive only excitatory input from the pyramidal neurons residing in the same cortical column. The pyramidal population receives an inhibitory input from the inhibitory interneurons, and an excitatory input [denoted with *p*^pyr^_tot, exc_(*t*)] coming from both the excitatory interneurons and from sources external to the column. The latter part we indicate with *p*^pyr^_ext, exc_(*t*), which for a cortical column *i* is given by:

(6) $$p_{\text{ext},\text{exc}}^{\text{pyr},i}(t)=p_{\text{const}}+\sum_{j}p_{\text{coup}}^{j,i}(t)+p_{\text{osc}}(t)+\xi_{\text{ou}}^{i}(t).$$

Here *p*_const_ is a constant component and ξ^*i*^_ou_(*t*) is a stochastic one. Those two components stand for a contribution from sensory input or brain areas that are not explicitly included in the model. The component ∑_*j*_ *p*^*j*, *i*^_coup_(*t*) is the summed contribution from other cortical columns (indexed with *j*) connected with column *i*, and the oscillatory component *p*_osc_(*t*) = *Ã* sin (2π*ft*) stands for a periodic stimulus.

The system of equations that describe the dynamics of a single cortical column *i* is given by:

(7,8,9) $$\left\{ \begin{array}{l} {\ddot{y}}_{0}^{i}(t)+2a{\dot{y}}_{0}^{i}(t)+a^{2}y_{0}^{i}(t)=Aa\text{ Sigm}[y_{1}^{i}(t)-y_{2}^{i}(t)]\\ {\ddot{y}}_{1}^{i}(t)+2a{\dot{y}}_{1}^{i}(t)+a^{2}y_{1}^{i}(t)=Aa\{p_{\text{ext},\text{exc}}^{\text{pyr},i}(t)\\ \;+C_{2}\text{ Sigm}[C_{1}y_{0}^{i}(t)]\}\\ {\ddot{y}}_{2}^{i}(t)+2b{\dot{y}}_{2}^{i}(t)+b^{2}y_{2}^{i}(t)=Bb\{C_{4}\text{ Sigm}[C_{3}y_{0}^{i}(t)]\} \end{array}\right.$$

where *i* runs from 1 to the number of columns *N*. *y*_0_ is the excitatory postsynaptic membrane potential that feeds into the two populations of interneurons and *y*_1_ and *y*_2_ are excitatory and inhibitory postsynaptic membrane potentials that enter into the pyramidal population, respectively. *C_k_* (*k* = 1, 2, 3, 4) are constants representing the connection strengths between populations.

The pyramidal neurons are known to be the main source of the EEG signal, which locally is proportional to the difference between their excitatory and inhibitory potentials. In the notation introduced above this value is expressed as *y*_1_(*t*) − *y*_2_(*t*). Below we will analyze the model behavior in terms of this quantity, in order to compare it with experimental data. Model parameters are listed in Table 1.

**Table 1:** **Parameter values of our neural mass model**.

**Variable**	**Symbol**	**Value**	**Units**
Number of columns	*N*	4, unless stated otherwise	–
Integration step	*h*	0.001	s
Noise intensity	*D*	350, unless stated otherwise	Hz
Noise correlation time	τ	0.15, unless stated otherwise	s
Constant input component	*p*_const_	75 for coupled system, 90 for uncoupled	Hz
Coupling strength between the columns	*K*	15, unless stated otherwise	–
Driving sine signal frequency	*f*	0.25, unless stated otherwise	Hz
Driving sine signal amplitude	*Ã*	45, unless stated otherwise	Hz
Composed signal amplitude	*Ã*′	10.76	Hz
Composed signal minimal frequency	*f*_min_	0.05	Hz
Composed signal maximal frequency	*f*_max_	4	Hz
Composed signal frequency step	*f*_step_	0.05	Hz
Length of simulation	–	1010	s
Length of rejected transient	–	10	s

*Parameters e_0_, v_0_, r, A, B, a, b, C_1_, C_2_, C_3_, C_4_ were set to plausible values as in Jansen and Rit (1995)*.

The Jansen-Rit model is capable of exhibiting two different types of periodic behavior: alpha oscillations and spiky oscillations (Grimbert and Faugeras, 2006; Spiegler et al., 2010). The latter are characterized by a lower frequency and higher amplitude than the alpha dynamics. The dynamical regime in which the system operates depends on the external excitatory input *p*^pyr^_ext, exc_. For the parameters used here and in the sole presence of the constant input component (*p*^pyr^_ext, exc_ = *p*_const_), the model is known (Grimbert and Faugeras, 2006) to undergo a Hopf bifurcation at an input value *p*_const_ = 89.83 Hz, above which a limit cycle appears that corresponds to the alpha oscillations. For 113.58 > *p*_const_ > 137.38 Hz, the alpha regime coexists with spiky oscillations, whereas for *p*_const_ > 137.38 Hz the alpha regime becomes the only attractor again. Finally, for *p*_const_ = 315.70 Hz the system undergoes another Hopf bifurcation and the limit cycle collapses back to a fixed point.

Experimental observations reveal a large autocorrelation time in EEG signals (see Aburn et al., 2012, and references therein). A number of studies interpret this observation as an instance of critical behavior such as the one found in the proximity of second-order phase transitions (Chialvo, 2010; Deco et al., 2013). This suggestion is, however, still under debate; for example Bédard et al. (2006) explained one of the putative signatures of criticality, 1/*f* scaling of the EEG power spectrum, in a way that does not rely on critical phenomena, but on filtering properties of the brain's tissue. In our simplified Jansen-Rit model description, we reproduce this spectral feature by operating close to one of the two Hopf bifurcations delimiting the alpha regime (Aburn et al., 2012). This allows us to see an emergent, but not fully developed, alpha resonance, characteristic of the proximity of a transition to an oscillatory regime (Kang et al., 2010; Battaglia and Hansel, 2011). We set the external excitatory input parameters in such a way that its value averaged over time (and over columns in case of the network of columns), 〈*p*^pyr^_ext, exc_〉 ≃ 90 Hz, is located close to the first Hopf bifurcation point. The external input *p*^pyr^_ext, exc_ delivered to each cortical column contains constant, stochastic and periodic components, as well as an input coming from coupled columns. Only the constant component *p*_const_ and the contribution from the afferent columns ∑*_j_ p*^*j*, *i*^_coup_(*t*) have non-zero mean, and therefore determine 〈*p*^pyr^_ext, exc_〉. The columns receive a common periodic signal mimicking a sensory stimulus received by cortical areas from the thalamus. Stochastic and periodic components have zero means and thus, even though they do not affect the average of the total input 〈*p*^pyr^_ext, exc_〉, they do contribute to its variance.

As mentioned in the Introduction, the brain contains sources of noise originating from different mechanisms (Faisal et al., 2008). Noise has been taken into account in past studies of the Jansen-Rit model (Jansen and Rit, 1995; Pons et al., 2010; Aburn et al., 2012) usually in the form of white noise. Here we use Ornstein-Uhlenbeck noise, which has a finite correlation time and is a more realistic representation of background synaptic noise. Destexhe and Rudolph (2004), for instance, showed that Poisson spike trains acting upon a neuron lead to a temporal correlation in membrane conductivity fluctuations, which under certain conditions can be modeled with Ornstein-Uhlenbeck noise. This noise, corresponding to ξ_ou_ variable in Equation (6), is generated by the following linear stochastic differential equation:

(10) $$\begin{array}{ll} \frac{d\xi_{\text{ou}}}{dt} & =-\frac{\xi_{\text{ou}}}{\tau }+\frac{\sqrt{2D}}{\tau }\xi_{w}(t) \end{array}$$

where ξ_*w*_(*t*) is a random variable representing Gaussian white noise with zero mean, 2*D* defines the amplitude of the stochastic component and τ is the correlation time of the Ornstein-Uhlenbeck noise. Each column in the model was fed with an independent realization of the Ornstein-Uhlenbeck noise.

### 2.2. Network of coupled cortical columns

The full model is composed of a number of cortical columns, each modeled in the way described above and connected with each other via the pyramidal neurons. For simplicity we choose all-to-all bidirectional connectivity and do not consider delay in the coupling (see Figure 1). The latter is justified by the fact that neighboring cortical columns are separated by a distance smaller than 1 mm and signal speed propagation in axons has a lower limit 0.1 m/s (Segev and Schneidman, 1999). Therefore, the delay between neighboring columns is not larger than milliseconds, which is at least one order of magnitude smaller than the characteristic timescales of the system. We performed simulation with non-zero time delays that reproduced qualitatively the results reported below, thereby validating this approach.

The input component ∑*_j_ p*^*j*, *i*^_coup_(*t*) in Equation (6) stands for the excitatory contribution to column *i* from its neighboring columns, and is given by:

(11) $$\begin{array}{l} \sum_{j}p_{\text{coup}}^{j,i}(t)=\frac{1}{N-1}\sum_{\begin{array}{c} j=1\\ j\neq i \end{array}}^{N}K_{j,i}\text{Sigm}[y_{1}^{j}(t)-y_{2}^{j}(t)], \end{array}$$

where the matrix 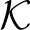 stands for the excitatory connectivity strengths between columns. The diagonal elements of 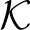 are equal to zero (no self-connectivity), and all other elements are equal to each other, taking a value that from now on will be referred to as the connectivity strength *K*. The total external contribution to a single cortical column is normalized by the number of its afferent connections. The contribution from one cortical column to the input of other columns is determined by the activity of the former, which depends on its own total input. So, the values of *p*_const_ and ∑*_j_ p*^*j*, *i*^_coup_ (through *K*) are chosen adequately in order to establish self consistent conditions that yield an effective input 〈*p*^pyr^_ext, exc_〉 ≃ 90 Hz.

### 2.3. Numerical methods

The model was integrated using the stochastic Heun integration method (Toral and Colet, 2014) with a time step equal to 0.001 s. We validated the integration method by running computations with decreased integration step. In each run we simulated 1010 s of activity, discarding the first 10 s. We computed the power spectra by applying the Welch algorithm from the Matplotlib Python module, using a Hanning window. The length of each time segment was chosen to be 20 s, with an overlap between segments equal to 10 s.

## 3. Results

### 3.1. Spectral and temporal properties of a single column

**Figure 2:**
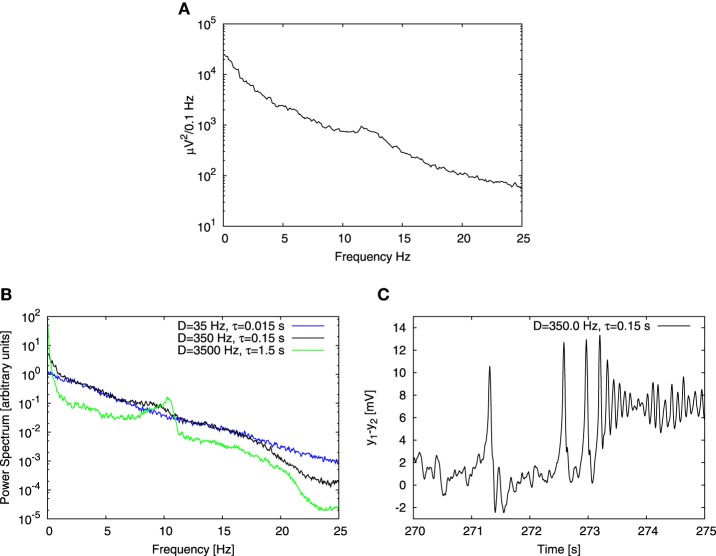
**Comparison of experimental and computational power spectra. (A)** Shows experimental data extracted from Figure 1D of the article by Bayer et al. (2011). **(B)** Shows power spectra obtained for three different noise parameter sets. Variance of the noise is the same for each case, but noise intensity *D* and correlation time τ change. Black line corresponds to time series shown in **(C)**, green line to conditions when the correlation time τ is 10 times increased and the blue one when it is 10 times decreased. **(C)** Shows the time trace obtained from a Jansen-Rit model of a single cortical column with an input consisting of two parts: a constant component equal to 90 Hz and a stochastic one determined by Ornstein-Uhlenbeck noise with intensity *D* = 350 Hz and correlation time τ = 0.15 s. Three distinct types of dynamics are apparent: time trace begins with a noisy behavior based on the fixed point, then spiky dynamics show up and finally time trace ends with alpha oscillations.

We first studied the behavior of a single cortical column receiving an input *p*_const_ = 90 Hz, systematically varying the parameters of the Ornstein-Uhlenbeck noise, namely its noise intensity *D* (varied in a range from 0.1 Hz to 1000 Hz) and correlation time τ (varied in a range from 0.001 s to 10 s). We obtained the power spectrum in each case and compared it with the one reported in the experiments of Bayer et al. (2011) (see Figure 2A). Our goal here was to choose the noise parameters for which the computational result reproduced the experimental characteristics, namely an 1/*f^b^* shape with an embedded peak in the alpha band. Figure 2B shows three power spectra obtained from the model driven by noisy inputs with the same variance but different intensities and correlation times. We found that experimental characteristics were qualitatively reproduced for τ = 0.15 s and noise intensity *D* = 350 Hz (see Figures 2A,B). Our result is robust for a range of *D* and τ values, provided that τ ≲ 0.2 s. Beyond that region, the power spectrum at low frequencies (≲ 2 Hz) becomes noticeably steeper than at higher frequencies (green trace in Figure 2B), which is not the case for experimental data. Moreover, for these large τ values the alpha peak becomes too prominent, whereas in the opposite limit it decreases as the correlation time τ is reduced (blue trace in Figure 2B), becoming for τ < 0.15 s significantly smaller (with respect to the 1/*f^b^* background) than in experimental data. This dependence of the signal spectrum on the noise characteristics can be explained in the following way: the system operates on average close to the Hopf bifurcation, where the limit cycle regime begins. This regime is explored transiently by the system due to the stochastic driving. The duration of the episodes in which the system stays in the oscillatory regime is dictated by the correlation time of the noise. Small τ implies rapid changes of the input to the system, which does not have time to relax in the limit cycle regime and alpha oscillations do not occur. In contrast, for relatively large τ, the input changes in a more smooth manner and the system has time to relax and exhibit alpha oscillations, which contribute to the alpha peak in the power spectrum. The noise intensity *D* plays a role too, because it dictates how deep the system can go into the limit cycle regime. The broadband shape of the power spectrum roughly follows the shape of the spectrum of the noise, which depends on the control parameter τ. This effect is noticeable specially for low frequencies, and originates in the regime which in deterministic conditions corresponds to a fixed point (referred to as a “random fixed point” from now on), where the system follows the noisy driving, and thus yields power spectra similar to that of the Ornstein-Uhlenbeck noise. In this way, the combined effect of different dynamics gives rise to a realistic power spectrum. This effect does not rely on critical behavior, but requires that the system explores different dynamical regimes.

The correlation time τ dictated by synaptic effects is conjectured to be of the order of 10 ms (Mazzoni et al., 2008; Sancristóbal et al., 2013) rather than 100 ms. In our case, however, noise stands for background activity arising from collective effects at the mesoscopic scale. In this study we focused on alpha rhythm, which has a characteristic time scale ~ 0.1 s, therefore choosing a noise correlation time τ equal to 0.15 s is reasonable. Note also that in the computational results shown in panel B of Figure 2 the alpha peak is shifted toward lower frequencies with respect to experimental results shown in panel A. The location of the peak could have been shifted by changing parameters of the model, however we chose to perform the analysis with the original set of parameters proposed by Jansen and Rit (1995), in order to maintain coherence with studies that adopted that set of parameters.

Although the power spectrum obtained with these noise parameters (Figure 2B) reproduces qualitatively the experimental results (Figure 2A), its corresponding temporal evolution exhibits a strong spiky behavior (Figure 2C), which is far from what is typically observed in experimental EEG recordings of healthy subjects. An adequate change of noise parameters would suppress the spiky dynamics in favor of alpha oscillations, but then the alpha peak in the power spectrum would be much higher than the 1/*f^b^* background, in contrast with the experimental observations. Therefore, we conclude that the behavior of a single column is not able to recapitulate realistically both the temporal and spectral characteristics of the experimental observations at the same time. For this reason we extended our model to several coupled columns.

### 3.2. Coupled cortical columns

**Figure 3:**
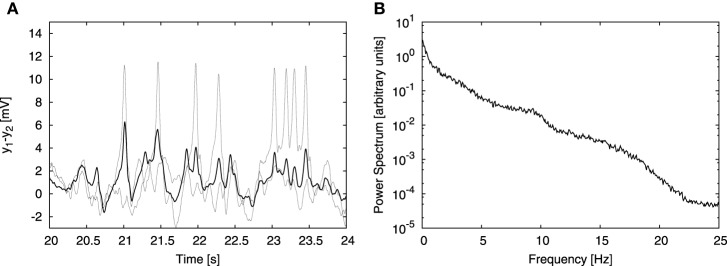
**Effect of signal averaging**. **(A, B)** Show a typical time trace and power spectrum, respectively, analogous to **(B, C)** of Figure 2, but obtained from the model of four columns coupled in an all-to-all bidirectional manner. The coupling strength constant *K* was set to 15. Besides the input from the other columns, each column received the same external input as in the single column case, described above. **(A)** Shows time traces of two individual columns (dashed lines) and the average signal of all four columns (solid line). Averaging attenuates individual spikes, but does not affect the power spectrum substantially (as shown in **B**).

The signals measured in experimental EEG recordings do not arise from a single cortical column, but from an aggregate of columns. In order to take this into account we extended our model to represent multiple coupled columns. As a simplifying assumption, we consider that the signal registered by an electrode is an average of the signals generated by individual columns in the probed area. The model considers only excitatory connections between populations of pyramidal neurons residing in different cortical columns. There are two new parameters with respect to the single-column case: the number of columns *N* and the coupling strength *K*. Our aim was to test our cross-frequency transfer hypothesis in a simple model, therefore we considered only *N* = 4 cortical columns coupled in a simple all-to-all manner with equally strong connections. Already for 4 coupled columns, individual spikes in the temporal domain are substantially attenuated due to averaging (Figure 3A), rendering time traces that qualitatively resemble EEG signals. On the other hand, the power spectrum of the averaged signal still resembles the experimental one (Figure 3B). This approach finds support in experiments; so-called “microseizures”—spiky, epileptic-like activity that may be detected only in a very fine spatial scale (~1 mm electrode array resolution, 40 μm electrode size)—have been observed experimentally by Stead et al. (2010), not only in epileptic subjects, but also sporadically in healthy ones.

The coupling contribution to each column was normalized by the number of afferent columns [*N* − 1 for our all-to-all connectivity topology, as expressed in Equation (11)]. This normalization allowed us to study the dependence of the results on the system size *N* for a constant value of the coupling strength *K*. Without that normalization, increasing the number of columns *N* in all-to-all topology would ultimately lead to saturation due to excessive external driving. Increasing the number of columns to *N* = 100 showed that the spectrum remains qualitatively consistent with the experimental one independently of the system size.

We next studied how robust the behavior shown in Figure 3 above is with respect to changes in the constant input and coupling strength. To vary those parameters it is necessary to take into account the fact that the input into a cortical column from other columns [∑*jp*^*j*, *i*^_coup_(*t*)] is implicitly dependent on *p*_const_, which has the same value for all columns and determines the dynamical regime in which the columns operate. In order to keep the effective total input 〈*p*^pyr, *i*^_ext, exc_〉 close to 90 Hz, the coupling strength *K* needs to be compensated by reduction in the input constant component *p*_const_ below 90 Hz. Taking this into consideration, we ran a series of simulations for *N* = 4 columns varying both the constant input component *p*_const_ and the coupling strength *K*. For each condition we averaged the inputs coming from the coupling terms over time and over all columns. In this manner we obtained the mean contribution of inter-column coupling to the input of a column. Adding this value to the constant input *p*_const_ gives the average total input acting upon a column, 〈*p*^pyr^_ext, exc_〉. We varied the coupling strength in the range 0 < *K* < 70, and in each case we chose the constant input component within the range 50 Hz < *p*_const_ < 90 Hz in such a way that the average external input to each column 〈*p*^pyr^_ext, exc_〉 was close to 90 Hz, in accordance with our assumption regarding proximity to the bifurcation point. In these conditions we found a linear dependence of the average of the coupling input on coupling strength *K*. Moreover, we found that the variation coefficient of this input was close to unity, which indicates that not only its average, but also its standard deviation grows linearly with *K*. This can be explained by two effects. First, higher synchronization causes in-sync spiking that weakens the effect of averaging between the columns. Second, these periods of high activity alternate with periods of low activity when due to the lower value of *p*_const_ the columns operate effectively in the fixed point regime.

In the whole parameter range that fulfills the condition 〈*p*^pyr^_ext, exc_〉 ~ 90 Hz, the temporal behavior and power spectrum resembled the experimentally observed one (results not shown). With that caveat, our results are robust not only with respect to the parameters of the Ornstein-Uhlenbeck noise and the system size, but also with respect to the constant input and coupling strengths. The simulations described below correspond to *K* = 15 and *p*_const_ = 75 Hz.

### 3.3. Effect of an oscillatory input

**Figure 4:**
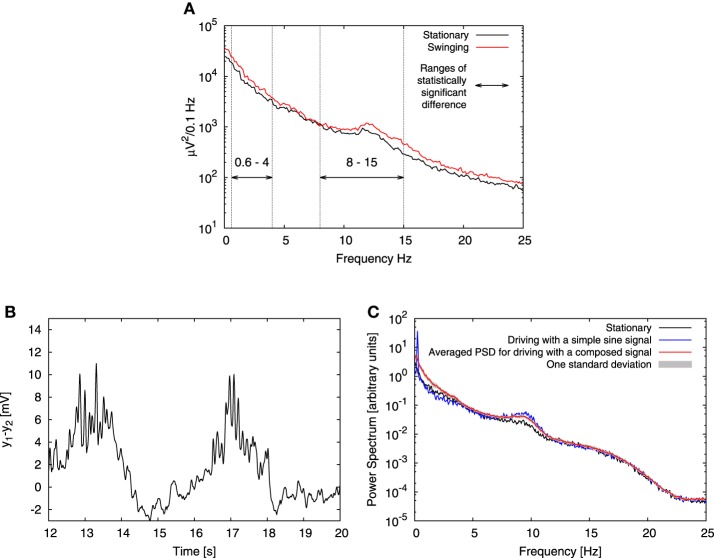
**Effect of low-frequency driving**. **(A)** Shows experimental EEG power spectra published by Bayer et al. (2011) (Figure 1D of that article), recorded from the Fz electrode in N2 sleep phase for both stationary (black line) and swinging (red line) conditions. Bayer et al. (2011) performed paired 2-tailed *t*-tests and found statistically significant increase in power in the ranges denoted here with horizontal arrows. In these ranges they found *p*-value to be *p* < 0.05, except for the frequency range 1 Hz–2 Hz, where *p* < 0.005. **(B)** Shows a typical time trace obtained from the model for two full periods of the driving sine signal, for which **(C)** shows the corresponding power spectra obtained in the periodically driven case (blue line) compared with the absence of driving (black line) and driving with the composed signal (red). This signal comprises sinusoid ingredients with varying frequencies, amplitudes and random phases. See text for details. Gray marks one standard deviation of distribution of power spectra obtained for different values of phases in the composed signal. In all cases four all-to-all connected columns subject to Ornstein-Uhlenbeck noise with intensity *D* = 350 Hz and correlation time τ = 0.15 s were used. In the case of sinusoidal driving, the input had amplitude *Ã* = 45 Hz and frequency *f* = 0.25 Hz. The columns were coupled with coupling strength *K* = 15.

The experimental study that we are interested in was performed by Bayer et al. (2011) on human subjects. Volunteers were placed on a bed, which was swung at a frequency 0.25 Hz. In our model we represent the stimulus associated with this movement as a harmonic driving. EEG data was recorded from the Fz electrode during the N2 sleep phase of the subjects for two conditions: swinging (bed in motion) and stationary (bed still). The experiment showed that swinging facilitates the transition from the awake state to sleep, and that it enhances the EEG power of both slow and alpha oscillations (see Figure 4A). According to the experimental setup the bed motion is harmonic, thus we started by mimicking swinging applying to each column in our model an oscillatory component *Ã* sin (2π*ft*), with *f* = 0.25 Hz. We set the driving amplitude *Ã* to 45 Hz, and left all other parameters unchanged with respect to the stationary conditions described in the previous section.

A typical time trace and power spectrum of the signal obtained from the model with harmonic driving are presented in Figures 4B,C. Panel B shows that both the instantaneous amplitude of the alpha oscillations and the average value of the signal are modulated by driving signal. The mechanism underlying both these effects originates in the bifurcation structure of the model (Grimbert and Faugeras, 2006; Spiegler et al., 2010), and was first reported by Tsodyks et al. (1997) for the case of gamma-theta coupling in a Wilson-Cowan model. Recently this effect was more generally discussed by Deco et al. (2008). We now examine in detail this mechanism for our case. In stationary conditions, the system explores four different dynamical regimes: two random fixed points, a regime of spiky behavior and a regime of alpha oscillations. This exploration arises as a consequence of the conjunction of two factors. The first factor is the proximity and coexistence of different dynamical regimes in the vicinity of the chosen input value 〈*p*^pyr^_ext, exc_〉 = 90 Hz. The second factor is stochastic driving, which enforces alternations between these regimes. For the noise parameters chosen here, *D* = 350 Hz and τ = 0.15 s, the variance of the Ornstein-Uhlenbeck noise is ≃ 48 Hz, which means that the system may explore two regimes separated by the Hopf bifurcation at 89.83 Hz: a regime of alpha oscillations and a random fixed point. Moreover, the system can enter the spiky dynamics regime, which starts at a saddle-node bifurcation at 113.58 Hz. Below this point the system may enter another random fixed point regime from which it may undergo noisy excitation and also exhibit spiky dynamics.

**Figure 5:**
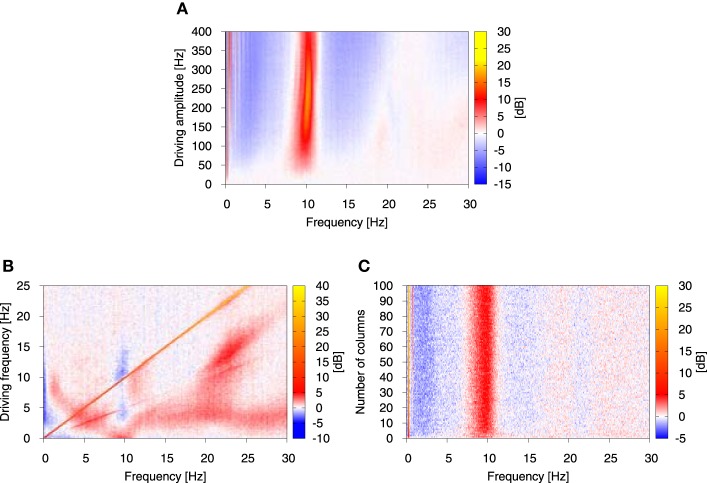
**Relative change of the power spectrum with respect to stationary conditions as the function of the amplitude (A) and frequency (B) of the driving oscillatory signal, and the number of columns in the system (C)**. Color represents the relative change of the power spectrum expressed in dB, as defined by 10 log_10_(PSD_driven_/PSD_stat_), where PSD_driven_ is the power spectrum in the presence of driving and PSD_stat_ is the power spectrum in its absence. In **(A, B)** the number of columns was *N* = 4, in **(B, C)** the driving amplitude *Ã* was fixed to 45 Hz and in **(A, C)** the driving frequency was fixed to 0.25 Hz. Again, the analysis was performed on the system of all-to-all connected Jansen-Rit models of cortical columns in the presence of Ornstein-Uhlenbeck noise with intensity *D* = 350 Hz and correlation time τ = 0.15 s. The coupling strength between the modules *K* was set to 15.

In the presence of oscillatory driving this situation changes and a number of factors contribute to an overall increase of power in the alpha band. Firstly, the amplitude of alpha oscillations is smaller in the direct vicinity of the Hopf bifurcation (*p*^pyr^_ext, exc_ ≳ 90 Hz), than for greater *p*^pyr^_ext, exc_ values, determined by the driving signal amplitude. Secondly, for *p*^pyr^_ext, exc_ > 137.38 Hz the alpha oscillations become the only allowed dynamics, so flipping between different regimes ceases to occur. This results not only in an increased alpha activity of each individual column, but also in an increase of synchronization between the columns. For sufficiently high driving amplitude columns go through transient in-phase synchronization periods, where the averaged amplitude of their alpha oscillations is greater than in the periods of unsynchronized alpha activity. These periods may occur for only some, or for all columns in the system, they may be terminated by noise and then may reappear due to coupling between the columns. A few peaks, which emerge due to synchronization between the columns during the driving sine upswing are shown in Figure 4B. They appear only for single or few oscillation cycles. It is due to the fact that for this case the driving amplitude is *Ã* = 45 Hz and thereby sets the maximal value of the deterministic part of the input to 135 Hz, which coincides with the starting point of the purely alpha regime at 137.38 Hz. This regime may still be explored due to noise, but under these conditions the system operates at best on the edge of resonance (Kang et al., 2010; Battaglia and Hansel, 2011). To the contrary, for higher driving amplitudes synchronization develops fully, enhancing further power increase in the alpha band, as shown in Figure 5A.

During the positive half of the driving cycle the alpha oscillations are superimposed to the oscillatory signal (see Figure 4B). During the negative half of the cycle the system moves further away from the oscillatory regimes and it may dwell in one of the random fixed point regimes. Consequently, less spiky behavior is observed, which results in a slight decrease of power in 0 Hz–5 Hz frequency band that corresponds to spiking (Faugeras et al., 2009). This decrease is shown in Figure 4C and in all panels of Figure 5. In the case of alpha oscillations, however, the system may remain in the regime of the random fixed point associated with the alpha limit cycle via the Hopf bifurcation. Noisy perturbations alter the system which may oscillate with alpha frequency. For the noise parameters chosen here (*D* = 350 Hz and τ = 0.15 s) the latter effect is minor. Nevertheless, all the discussed effects together lead to an overall increase of power in the alpha band in comparison to stationary conditions, as shown in Figure 4C, thereby reproducing qualitatively the experimental observation shown in Figure 4A.

The experiment also showed a statistically significant increase of power for low frequencies. The increase is also observed in the model, but is much more centered (peaked) at the driving frequency (0.25 Hz) than in the experiment, which is much smoother, probably due to reshaping of the low-frequency harmonic signal by sensory, thalamic and/or thalamocortical processing. In order to test this assumption, in the next step we drove the model with a reshaped signal of the form:

(12) $$f(t)=\tilde{A^{\prime }}\sum_{n=n_{\text{min}}}^{n_{\text{max}}}10^{-\frac{n\cdot f_{\text{step}}-f_{\text{min}}}{f_{\text{max}}-f_{\text{min}}}}\sin[2\pi (nf_{\text{step}}t+X_{n})]$$

where *n*_min_ = *f*_min_/*f*_step_, *n*_max_ = *f*_max_/*f*_step_, *f*_min_ = 0.05 Hz is the minimal frequency of the driving, *f*_max_ = 4 Hz is the maximal one, *f*_step_ was set to 0.05 Hz and *X_n_* is a random number in the range [0,1). This formula describes a signal composed of a sum of sines with frequencies from *f*_min_ to *f*_max_ taken every *f*_step_, with exponentially decaying amplitudes and with randomly distributed phases. The choice of *f*_max_ has been dictated by the upper limit of the frequency interval in which Bayer et al. (2011) observed a significant increase of power. The amplitude *Ã*′ = 10.76 Hz was set is such a way that this composed signal delivered the same power to the model as the previously used simple sine signal with amplitude *Ã* = 45 Hz. We performed 10 full simulations for different distributions of random phases and averaged the power spectra obtained. The resulting averaged spectrum along with one standard deviation of the power spectrum distribution is shown in Figure 4C. This figure shows that driving with the composed signal reproduces the experimental results better than driving with a simple sine: the increase in the alpha band is present regardless of the randomization of phases, and instead of a decrease of power for low frequencies (as observed in the simple sine driving case), an increase (similar to the experimental result) is observed.

Next, in order to examine systematically the response of this broadband oscillation to harmonic signals, we studied the response of our model for *N* = 4 columns to a variation in the amplitude and frequency of the driving. Figure 5A depicts the dependence of the power spectrum (in color code) on the driving signal amplitude, with red indicating an *increase* in power and blue representing a *decrease* with respect to the stationary conditions. This figure shows that the power increase in the alpha band is robust with respect to the driving amplitude, provided its value is large enough. The slight increase in the frequency that responds maximally, observed for large amplitude values, might be understood from the fact that the frequency of the limit cycle exhibited by the NMM increases slightly for increasing input to the columns (Spiegler et al., 2010).

More importantly, we examined the response of the model with *N* = 4 columns for a large range of signal frequencies, ranging from values much smaller than its intrinsic alpha frequency (as we have been discussing so far) all the way to much larger frequencies (up to 25 Hz). The results, shown in Figure 5B, reveal that an increase in alpha occurs only for low-enough driving frequencies (*f* ≲ 2 Hz). As the driving frequency increases, the initial response at alpha splits and leads to increase of power at frequencies smaller and larger than alpha. Interestingly, at this point the alpha band undergoes a decrease, rather than an increase, in power. The low-frequency power (≲ 0.5 Hz) is also reduced for a wide range of driving frequencies. The response is dominated by a straight diagonal line corresponding to 1:1 response to the driving frequency, and by its first harmonic. This strong 1:1 response means that in the case of harmonic driving every injected frequency is transferred by the system. This explains why driving of the form as in Equation (12) leads to a broad increase of power in low frequencies in the system's output. The same study performed for stronger driving showed that the effects discussed above are robust with respect to the driving amplitude (results not shown), although for stronger signals higher harmonics show up and relative changes in the power spectrum are enhanced and widened. Finally, we studied the impact of the system size on the observed effect. The result shown in Figure 5C indicates that under the chosen conditions (oscillatory driving with amplitude *Ã* = 45 Hz and frequency *f* = 0.25 Hz) the results are robust with respect to the system size.

## 4. Discussion

We have studied a minimal model that gives rise to broadband oscillations in the alpha regime. The model consists of a small number of cortical columns coupled in an all-to-all configuration. Similarly to what happens in microscopic models (Sancristóbal et al., 2013), taking into account that the neuronal population receives a background signal from the rest of the brain in the form of a temporally correlated Ornstein-Uhlenbeck noise (Destexhe and Rudolph, 2004) leads to the characteristic 1/*f^b^* spectral profile of neural activity. Furthermore, selecting adequately the population dynamics of the NMM produces oscillations with a well-defined frequency (David and Friston, 2003), which appear superimposed to the 1/*f^b^* profile. On the other hand, generating this profile entails that the NMM operates in a spiking regime, which differs from the characteristic dynamics observed via EEG. This type of macroscopic measurement, however, reflects the behavior of multiple coupled columns, and we showed that when this situation is considered in our model the spiking behavior disappears due to averaging, rendering signals which recapitulate the experimentally observed EEG while maintaining the broad power spectrum.

Within this dynamical regime, we have examined the effect of a low-frequency driving with a simple sine signal and a composed one, showing a cross-frequency transfer through which this driving signal increases the power not only of low-frequency rhythms, but also of the alpha activity. The result qualitatively reproduces the experimental observations of Bayer et al. (2011) on the effect of rocking on alpha activity and sleep and is robust with respect to the choice of model parameters. The increase of power in the alpha band results from both an enhancement of alpha activity of individual cortical columns and collective synchronization effects. Our results suggest that certain types of cross-frequency transfer in the brain can be simply the result of passive driving of a broadband neuronal oscillator, which brings this effect close to the vast body of work dealing with the driving and synchronization of chaotic oscillators (Pikovsky et al., 2001; Boccaletti et al., 2002; Anishchenko et al., 2007) Interestingly, a systematic analysis shows that the frequency transfer only occurs toward the intrinsic frequency of the oscillator (alpha) when the driving frequency is low; as it increases the response shifts to both lower and higher frequencies, and the power in the alpha band decreases instead of increasing. Taken together, our results indicate that a relatively simple oscillation generation mechanism in neuronal populations has a strongly nontrivial response to periodic driving, providing a rich scenario to interpret a variety of cross-frequency phenomena in the brain.

### Conflict of interest statement

The authors declare that the research was conducted in the absence of any commercial or financial relationships that could be construed as a potential conflict of interest.
